# Low consumption of fruits and vegetables among adults in Uganda: findings from a countrywide cross-sectional survey

**DOI:** 10.1186/s13690-019-0332-6

**Published:** 2019-02-07

**Authors:** Steven Ndugwa Kabwama, Silver K. Bahendeka, Ronald Wesonga, Gerald Mutungi, David Guwatudde

**Affiliations:** 1 Makerere University School of Public Health, Kampala, Uganda; 2 St. Francis Hospital, Nsambya, Kampala, Uganda; 3 School of Statistics and Planning, Makerere University College of Business and Management Sciences, Kampala, Uganda; 4 Control of Non-Communicable Diseases Desk, Ministry of Health, Kampala, Uganda; 5 Department of Epidemiology & Biostatistics, School of Public Health, Makerere University College of Health Sciences, Kampala, Uganda

**Keywords:** Fruit and vegetable, Diet, Nutrition, WHO STEPs methodology, Sub-Saharan Africa, Uganda

## Abstract

**Introduction:**

Adequate consumption of fruits and vegetables has protective benefits against development of coronary heart disease, hypertension and chronic obstructive pulmonary disease. However, approximately 2.7 million deaths annually can be attributed to inadequate fruit and vegetable consumption. We analyzed data from a countrywide survey in Uganda, to estimate the prevalence of adequate fruit and/ or vegetable consumption, and identify associated factors.

**Methods:**

Data were collected using the World Health Organization STEPwise approach to surveillance, a standard approach to surveillance of risk factors for Non Communicable Diseases. Fruit and vegetable consumption was assessed by asking participants the number of days in a typical week they eat fruits or vegetables and the number of servings eaten in one of those days. Adequate fruit and/ or vegetable consumption was defined as consuming 5 or more servings of fruits and/ or vegetables per day in a typical week. We used modified Poisson regression analysis to estimate prevalence risk ratios (PRRs) and identify factors associated with eating 5 or more servings of fruits and/ or vegetables per day, per week.

**Results:**

Of 3962 participants, 484 (12.2%) consumed 5 or more servings of fruits and/ or vegetables per day in a typical week. Participants who were married or cohabiting were more likely to consume at least 5 servings of fruits and/ or vegetables per day in a typical week compared with those who had never been married PRR = 1.51 [95% CI 1.07–2.14]. Compared with participants from Western region, those from Central region were more likely to consume 5 or more servings of fruits and/ or vegetables per day in a typical week, PRR = 3.54 [95% CI 2.46–5.10] as were those from Northern, PRR = 2.90 [95% CI 2.00–4.23] and Eastern regions PRR = 1.60 [95% CI 1.04–2.47].

**Conclusions:**

Fruit and vegetable consumption in Uganda is low and does not differ significantly across social and demographic characteristics, except marital status and geographical region of residence. There is a need to develop and strengthen policies that promote adequate consumption of fruits and vegetables in the Ugandan population.

## Introduction

According to the World Health Organization (WHO), approximately 2.7 million deaths annually can be attributed to inadequate consumption of fruits and vegetables [1]. This figure includes 11% of strokes and 31% of ischemic heart diseases worldwide. A systematic review and meta-analysis of prospective studies investigating the association between fruit and vegetable consumption and all-cause mortality established that the average reduction of mortality risk was 5% for each additional serving of fruits and vegetables per day [2]. More specifically, consumption of fruits and vegetables has been shown to have protective benefits against development of coronary heart disease, hypertension and chronic obstructive pulmonary disease [3]. Although the benefits of consuming fruits and vegetables are well documented, a World Health Survey that compared fruit and vegetable consumption across 52 low and middle income countries found that only about 30% of men and women consumed more than the recommended minimum of 5 servings of fruits and/ or vegetables every day [4]. Fruits and vegetables are an important source of vitamins, vital minerals and dietary fiber which in itself has been associated with lower incidence of obesity [5]. In spite of the importance of consuming fruits and vegetables to human health, there is scanty information regarding the levels and adequacy of consumption of fruits and vegetables in Uganda.

In 2014, a countrywide survey was conducted to provide baseline estimates of the 4 major risk factors for non-communicable diseases (NCDs) including physical activity, alcohol use, tobacco use, as well as fruits and vegetables consumption among adults in Uganda. We analyzed data from this survey to provide national level estimates of the consumption of fruits and vegetables among adults in Uganda. Findings from the analysis can be used to inform development of comprehensive nutrition interventions and the implementation of the Uganda National Food and Nutrition Policy [6].

## Methods

### Study design

The Uganda National NCD Risk Factor Survey was conducted using a cross sectional study design, and data were collected between March and July 2014. The survey used the World Health Organization’s (WHO) STEPwise approach to surveillance, standardized method of collecting data on risk factors for NCDs [7]. Details about the conduct of the survey, including the sample size calculation and sampling procedures have all been described in previous publications [8–10]. We give a brief description of the sampling and methods used.

### Sampling

The survey utilized a three stage sampling design to obtain a sample that is representative of the national population. In the first stage, 350/ 78,950 enumeration areas were randomly selected from the Uganda Bureau of Statistics sampling frame of enumeration areas that was used during the 2014 population and housing census using probability proportionate to size sampling and stratified by the four geographical regions. The enumeration areas were selected with separate estimates for urban and rural areas. Urban areas were those classified as such by government designation or with a population density higher than 1000 people per square km. In the second stage, all households in each of the 350 enumeration areas were listed and 14 households were randomly selected. In the final stage, a member of the household aged 18–69 years was randomly selected to participate in the survey.

### Measurements

Data were collected in 3 steps in accordance with the standard WHO STEPwise approach to Surveillance questionnaire [7] as follows: In STEP 1, data were collected on socio-demographic and behavioral characteristics including alcohol and tobacco use, fruit and vegetable consumption and level of physical activity (PA). Fruits and vegetables consumption was assessed by asking participants the number of days in a typical week when they eat fruits or vegetables; and when they do, the number of servings of fruit or vegetables eaten in one of those days. A show card was used to show participants pictures of examples of commonly available fruits and vegetables in Uganda, with each picture representing the size of one serving. Participants were also asked whether within the past 3 years they had received lifestyle advice from a medical worker or other health worker to eat at least 5 servings of fruits and/ or vegetables each day.

In STEP 2, physical measurements including body height, body weight and blood pressure were made. Finally in STEP 3, a blood sample was collected to assess fasting plasma glucose using a CardioChek® PA meter [8, 10].

Data collection was conducted over 2 days whereby the interview (STEP 1) and physical measurements (STEP 2) were done on the first day while the biochemical measurements (STEP 3) were conducted the next day among participants reporting compliance with an overnight 8-h fast, no exercise or smoking.

### Statistical analysis

Due to their low caloric content and high fiber and nutrient density, the recommended level of consumption of fruits and/ or vegetables is a minimum of 5 servings per day [4, 11, 12]. Based on this recommendation, we created a binary variable of those who reported consuming less than 5 servings of fruits and/ or vegetables (including those who did not eat any per week); and those who reported consuming at least 5 servings. We then used weighted modified Poisson regression modeling to identify characteristics of the participants associated with consuming at least 5 servings of fruits and/ or vegetables, with the participant sampling selection fractions as the weights. Weighted modified Poisson regression was preferred over logistic regression to avoid under estimation of the standard errors of the estimated risk ratios [13–15]. Thus we estimated both the crude and adjusted prevalence risk ratios (PRRs) and their corresponding 95% confidence intervals (CIs) as the measure of association between the binary outcome of consuming at least 5 servings of fruits and vegetables per day in a typical week, or not. Model fitting was conducted first including in the model, all social, demographic, and physical characteristic variables of the participants measured. Then backward stepwise elimination was used to remove variables not significantly associated with the outcome. Variables were removed one at a time, starting with the least significantly associated with the outcome. The cut-off for statistical significance was a *p*-value of < 0.05. Therefore, variables with a p-value ≥0.05 were removed from the model. The independent variables investigated during modelling included: sex, age, level of education, marital status, geographical region of residence, employment status, urban or rural residence, Body Mass Index (BMI), blood pressure and Fasting Plasma Glucose (FPG) level, having received lifestyle advice to consume at least 5 servings of fruits and vegetables per day. When building a multiple regression model, including variables that are highly correlated affects the integrity of the model leading to wrong estimates of the standard errors [16]. We thus investigated multicollinearity in the regression model using the variance inflation factor where by a mean variance inflation factor less than 10 showed that there was no multicollinearity. The pairs of variables investigated include level of education and urban/ rural residence, employment status and level of education and urban/ rural residence and employment status. All statistical analyses were performed using STATA version 12 (StataCorp, College Station, Texas, USA).

## Results

### Characteristics of participants

Of the targeted study sample of 4100, 3987 consented to participate, yielding a response rate of 97%. Among these, 3962 had data on fruit and vegetable consumption and are therefore included in this analysis, of which 2371 (59.8%) were females, 2886 (72.8%) were rural residents, 2634 (66.4%) were either married or cohabiting, and 2585 (65.2%) were in employment (Table 1).

**Table 1:** Characteristics of participants in the survey

Characteristic	-n- (*n* = 3962)	Summary measure (%)
Sex		
Male	1591	40.2
Female	2371	59.8
Age group, years		
18–29	1610	40.6
30–49	1666	42.1
50–69	686	17.3
Residence		
Urban	1076	27.2
Rural	2886	72.8
Region		
Western	940	23.7
Central	962	24.3
Northern	778	19.6
Eastern	1282	32.4
Ethnicity		
Baganda	768	19.4
Banyankore/ Bakiga	698	17.6
Basoga	315	7.9
Banyoro/ Batooro	257	6.5
Lugbara/ Madi	249	6.3
Other	1675	42.3
Marital status		
Never married	623	15.7
Married/ Cohabiting	2634	66.4
Separated/ Divorced/ Widowed	702	17.8
Completed level of education		
No formal school	649	15.7
Primary school	1614	40.7
Secondary school	1310	33.1
University or higher	374	9.4
Employment status		
Employed	2585	65.2
Unemployed	1376	34.7
BMI^a^ (kg/m^2^)		
Underweight, < 18.5	328	8.3
Normal weight, 18.5–24.9	2518	63.6
Overweight or Obese, ≥25	1116	28.2

^a^Body Mass Index

### Levels of fruit and vegetable consumption

Among the participants in the survey, 484 (12.2%) reported consuming 5 or more servings of fruits and/ or vegetables per day in a typical week. Among participants who had never been married, 560 (89.9%) consumed < 5 servings of fruits and/ or vegetables in a day in a typical week while 63 (10.1%) consumed ≥5 servings in a day in a typical week. Among those who were married on the other hand, 2298 (87.2%) consumed < 5 servings of fruits and/ or vegetables a day in a typical week and 336 (12.8%) consumed ≥5 servings of fruits and/ or vegetables a day in a typical week. The distribution of consumption of the different servings of fruits and vegetables is summarized in Table 2.

**Table 2:** Distribution of number of servings of fruits and/ or vegetables eaten per day in a typical week, by characteristics of participants

Variable	-n-	Servings of fruits and/ or vegetables consumed		*p*-value
		< 5 servings *n* (%)	≥ 5 servings *n* (%)	
All participants	3962	3478 (87.8)	484 (12.2)	n/a
Sex				
Males	1591	1384 (87.0)	207 (13.0)	0.21
Females	2371	2094 (88.3)	277 (11.7)	
Age				
18–29	1610	1432 (88.9)	178 (11.1)	0.17
30–49	1666	1452 (87.2)	214 (12.8)	
50–69	686	594 (86.6)	92 (13.4)	
Marital status				
Never married	623	560 (89.9)	63 (10.1)	0.19
Married/Cohabiting	2634	2298 (87.2)	336 (12.8)	
Separated/Divorced/ Widowed	702	617 (87.9)	85 (12.1)	
Level of education				
No formal schooling	649	566 (87.2)	83 (12.8)	0.70
Primary	1614	1408 (87.2)	206 (12.8)	
Secondary	1310	1158 (88.4)	152 (11.6)	
University or higher	374	332 (88.8)	42 (11.2)	
Region of residence				
Western	940	890 (94.7)	50 (5.3)	< 0.05
Central	962	766 (79.6)	196 (20.4)	
Northern	778	636 (81.7)	142 (18.3)	
Eastern	1282	1186 (92.5)	96 (7.5)	
Rural-urban residence				
Urban	1076	976 (90.7)	100 (9.3)	< 0.05
Rural	2886	2502 (86.2)	384 (13.8)	
Employment				
Employed	2585	2283 (87.8)	302 (12.2)	0.16
Unemployed	1376	1194 (86.7)	182 (13.3)	
BMI (kg/m^2^)				
18.5–24.9 Normal weight	2518	2201 (87.4)	317 (12.6)	0.40
< 18.5 Underweight	328	285 (87.0)	43 (13.0)	
≥ 25 Overweight or Obese	1116	992 (88.9)	124 (11.1)	

### Factors associated with fruits and/ or vegetable consumption

The three pairs of variables investigated for multicollinearity yielded mean variance inflation factors less than 10 meaning that there was no multicollinearity in the regression model.

Only marital status and geographical region of residence were the factors found to be statistically significantly associated with eating 5 or more servings of fruits and/ or vegetables per day in a typical week. Participants who were married or cohabiting were more likely to consume 5 or more servings of fruits and/ or vegetables per day in a typical week compared with those who had never been married, PRR = 1.51 [95% CI 1.07–2.14]. Further, compared with participants resident in the Western region of the country, those resident in the Central region were more likely to consume 5 or more servings of fruits and/ or vegetables per day in a typical week, PRR = 3.54 [95% CI 2.46–5.10]; as were residents in the Northern, PRR = 2.90 [95% CI 2.00–4.23], and Eastern regions of the country, PRR = 1.60 [95% CI 1.04–2.47]. The rest of the results from association analysis are presented in Table 3.

**Table 3:** Crude and Adjusted Prevalence Risk Ratios (PRR) of consuming ≥5 servings of fruits and/ or vegetables per day in a typical week among participants in the survey

Variable	-*n*-	≥ 5 servings *n* (%)	Crude PRR [95% CI]	Adjusted PRR [95% CI]^a^
Sex				
Males	1591	207 (13.0)	1.0	1.0
Females	2371	277 (11.7)	0.82 [0.64–1.05]	0.82 [0.64–1.04]
Age				
18–29	1610	178 (11.1)	1.0	1.0
30–49	1666	214 (12.8)	1.11 [0.84–1.47]	1.15 [0.87–1.52]
50–69	686	92 (13.4)	1.03 [0.74–1.45]	1.20 [0.86–1.67]
Marital status				
Never married	623	63 (10.1)	1.0	1.0
Married/Cohabiting	2634	336 (12.8)	1.52 [1.04–2.21]	1.51 [1.07–2.14]
Separated/Divorced/ Widowed	702	85 (12.1)	1.38 [0.88–2.17]	1.46 [0.98–2.17]
Level of education				
No formal schooling	649	83 (12.8)	1.0	1.0
Primary	1614	206 (12.8)	0.86 [0.63–1.16]	0.91 [0.67–1.22]
Secondary	1310	152 (11.6)	0.98 [0.68–1.41]	1.06 [0.76–1.47]
University or higher	374	42 (11.2)	0.76 [0.47–1.22]	0.84 [0.54–1.31]
Region of residence				
Western	940	50 (5.3)	1.0	1.0
Central	962	196 (20.4)	3.54 [2.40–5.22]	3.54 [2.46–5.10]
Northern	778	142 (18.3)	2.83 [1.88–4.27]	2.90 [2.00–4.23]
Eastern	1282	96 (7.5)	1.45 [0.92–2.27]	1.60 [1.04–2.47]
Rural-urban residence				
Urban	1076	100 (9.3)	1.0	1.0
Rural	2886	384 (13.8)	1.00 [0.74–1.35]	0.99 [0.75–1.30]
Employment				
Employed	2585	302 (12.2)	1.0	1.0
Unemployed	1376	182 (13.3)	0.99 [0.74–1.32]	0.92 [0.70–1.22]
Received lifestyle advice on fruit and/ or vegetable consumption				
No	2916	359 (12.3)	1.0	1.0
Yes	890	123 (13.8)	1.21 [0.92–1.60]	1.18 [0.90–1.54]
BMI (kg/m^2^)				
18.5–24.9 Normal weight	2518	317 (12.6)	1.0	1.0
< 18.5 Underweight	328	43 (13.0)	0.88 [0.61–1.26]	0.86 [0.60–1.22]
≥ 25 Overweight or Obese	1116	124 (11.1)	1.03 [0.78–1.37]	0.98 [0.75–1.28]

^a^Adjusted for marital status and geographical region of residence

## Discussion

The analysis of data from this countrywide survey has revealed that the majority of adults in Uganda do not meet the recommended minimum requirements for fruit and vegetable consumption, with just over 1 in 10 adults meeting the recommended minimum of 5 or more servings of fruits and/ or vegetables per day in a typical week. Other countries in the region such as the Democratic Republic of Congo have also reported a closely low prevalence at 12.1% [17], whereas others such as the Republic of Tanzania, Zambia and Mozambique have reported much lower prevalence at 2.8, 3 and 5% respectively [18–20]. The low prevalence of adequate consumption of fruits and/ or vegetables in Sub-Saharan Africa has been attributed to variations in the availability of the fruits and vegetables in the region [21]. Indeed, the availability of fruits and vegetables per person per year is 105.5 kg in the African region and 64.2 kg per person per year in Uganda, way below the 146 kg per person per year recommended by the World Health Organization and the Food and Agriculture Organization [1]. The low consumption of fruits and vegetables has also been explained by cultural dietary patterns and an increasing urbanization rate in Sub-Saharan Africa [21]. In Uganda, the low availability of fruits and vegetables could be attributed to the limited knowledge about their nutritional value and the emphasis of cultivating commercial agricultural products such as roots/ tubers and cereals from which farmers earn income [22]. A health in all policies approach [23] should be adopted in the design and formulation of policies that promote income generation and poverty reduction so that health and nutrition are promoted in parallel with interventions that spur economic development.

The factors found significantly associated with adequate consumption of fruits and/ or vegetables were marital status and geographical region of residence. Previously published literature has shown that age [24], gender [24, 25], level of education [24–26] and knowledge about fruits and vegetables [27] are significant predictors of adequate fruit and vegetable consumption. Our analysis did not find these to be significantly associated with fruit and vegetable consumption. This is likely to be because the level of fruit and vegetable consumption is about the same in the Ugandan context, with the population differing by only a few demographic variables. Participants who were married were more likely to consume 5 or more servings of fruits and/ or vegetables in a typical week. The association between marital status and fruits and vegetable consumption where married people consume more fruits and vegetables compared with their unmarried counterparts is no surprise finding and has been documented in other studies [28–30]. Marriage and companionship involve social interactions which set the stage for higher food consumption as a result of a regular pattern of meals [30] that include fruits and vegetables. This observation could be explained by the theoretical model of social integration and social control where by marriage is associated with efforts to control health behavior among spouses [30, 31]. This control of health behavior could involve a spouse preparing meals rich in fruits and vegetables or ensuring the availability of the fruits and vegetables in the home.

Participants from some regions of residence in the county were statistically significantly more likely to consume the recommended 5 or more servings of fruits and/ or vegetables per day in a typical week compared with those from the Western region. This finding is corroborated by results from a food consumption survey that established that there are substantial variations across the geographical regions in Uganda regarding food and micronutrient consumption [32]. The diets in the various regions are based on locally available and commonly cultivated staples that are presumed to be nutrient dense [33]. Each geographical region has certain ethnicities part of whose cultural identities are the traditional staples consumed in that region [34]. Thus our findings regarding these regional differences are not surprising. Previous reports have also documented poor nutrition among people from the western region of the country [35–37]. A survey carried out in Western Uganda found that about 7 in 10 adults in the region have only 2 meals a day [38] such that food choices and dietary patterns are based on their energy and caloric content and not the variety in nutrient composition. Indeed, the Uganda National Household Survey 2016/2017 showed that in the week that preceded the survey, the Western region reported the highest prevalence of low dietary diversity where 43% of households reported to have consumed fewer than 5 of the 7 food groups (cereals/ tubers, pulses/ nuts, fruits, vegetables, milk, meat/ fish/ eggs and oil) [39]. Policies and interventions to improve nutritional status and enhance fruit and vegetable consumption in the country should take into account the cultural diversity across the geographical regions and its influence on food choices.

### Limitations

There are limitations to the interpretation of the findings from the analysis. The dependent variable where participants reported the number of days in a typical week when they eat fruits or vegetables and the number of servings of fruit or vegetables consumed in one of those days was based on self-reports which introduces information bias. The use of a nutrition card to aid participants in estimating the size of a serving does reduce but cannot totally eliminate this bias. This challenge of using self-reports in diet and nutritional epidemiology to assess food consumption in terms of quantity and frequency is not new and has been documented and findings critiqued in previous literature [40]. However, there is also a substantial body of evidence to show that there are no significant differences between estimates of nutrient intake using weighed records and recall questionnaires [41] as is the case with the recall of the number of servings of fruits and vegetables that was used in the current paper. Furthermore, even though the validity of the individual survey question on fruit and vegetable consumption remains to be validated in the study population, the validity of other forms of recall measures of fruit consumption is supported in the European population by the Bingham study [41]. Vegetable intake however remains uncertain. Thus the presence of this bias could have affected the validity of the results. We also acknowledge the possibility of a selection bias that could have been introduced because the data collection was carried out during the day at times when men are more likely to be away from home which could explain the disproportionately higher number of female participants compared with males. Indeed, the prevalence of consumption of adequate servings of fruits and vegetables was higher among males (13.0%) compared with females (11.7%) because of the larger denominator which could have biased the results away from the null. Also, in the assessment of fruit and/ or vegetable intake, participants were asked the number of days in a typical week when they eat fruits or vegetables; and when they do, the number of servings of fruit or vegetables eaten in one of those days. The word ‘typical’ is vague and its interpretation was left to the interviewee which could have introduced bias.

## Conclusions

The results from this country-wide survey have shown that there is low fruit and vegetable consumption in Uganda. There is a need to better understand the level of people’s understanding of fruits and vegetables and whether nutritional education may be an appropriate focus for the improvement of dietary behaviors among adults in Uganda. The design and development of policies to promote agricultural related socio-economic development should take into account the influence that these policies have on food options and choices. Fruit and vegetable consumption was only associated with geographical region of residence and marital status which may be a reflection of cultures in which food choices are part of cultural and ethnic identities. Policies and interventions to improve nutritional status and enhance fruit and vegetable consumption in the country should take into account the cultural diversity across the geographical regions and its influence on food choices.

## Abbreviations

BMI: Body Mass Index
STEPS: Stepwise Approach to Surveillance
WHO: World Health Organization
